# Stability, neurology, infection, and morbidity (SNIM): The quest towards a unified framework for assessment and management of spinal infection:A narrative review and position paper of the EANS spondylodiscitis study group

**DOI:** 10.1016/j.bas.2025.105636

**Published:** 2025-10-17

**Authors:** Jonathan Neuhoff, Santhosh G. Thavarajasingam, Ehab Shiban, Andreas K. Demetriades, Frank Kandziora, Florian Ringel, Benjamin Davies, Andreas Kramer

**Affiliations:** a Center for Spinal Surgery and Neurosurgery, Berufsgenossenschaftliche Unfallklinik Frankfurt am Main, Germany; b Department of Neurosurgery, LMU University Hospital, Munich, Germany; c Imperial Brain & Spine Initiative, Imperial College London, London, United Kingdom; d Department of Neurosurgery, Medical University Cottbus, Germany; e Edinburgh Spinal Surgery Outcome Studies Group, Department of Neurosurgery, Royal Infirmary Edinburgh, NHS Lothian, Edinburgh, United Kingdom; f Department of Academic Neurosurgery, Addenbroke's Hospital, Cambridge University Hospital NHS Healthcare Trust, Cambridge, United Kingdom; g Spondylodiscitis Study Group, EANS Spine Section, United Kingdom

## Abstract

**Background:**

Primary spinal infections - including spondylodiscitis, vertebral osteomyelitis and epidural abscess - are increasingly prevalent and clinically heterogeneous, yet no standardized assessment or treatment guidelines currently exist. This position paper proposes a structured framework to support clinical evaluation and management and to guide future trials.

**Major findings:**

Based on a comprehensive literature review and expert consensus, the authors identify four core domains essential for spinal infection evaluation: Stability, Neurology, Infection, and Morbidity (SNIM). Stability is assessed through clinical and radiographic indicators such as vertebral destruction, deformity, and pain. Neurological deficits—including radicular weakness or myelopathy—remain key indications for decompression, supported by imaging of neural compression. Infection control depends on accurate pathogen identification, targeted antimicrobial therapy, and, when indicated, surgical debridement or abscess drainage. Morbidity, including age, frailty and comorbidities, modifies treatment strategies and influences conservative and surgical outcomes, necessitating tailored, risk-adapted approaches. Building on these domains, the paper formulates testable hypotheses related to surgical interventions and introduces a conceptual treatment algorithm designed to reduce clinical heterogeneity and inform prospective study design.

**Conclusion:**

The SNIM framework is presented as a preliminary model intended to harmonize terminology, support trial development, and structure future research. While not a validated classification system, it provides a foundation for stratifying patients and exploring outcome-relevant variables. The proposed algorithm reflects current expert interpretation and is offered as a hypothesis-generating tool to be refined through collaborative validation efforts. This paper aims to stimulate ongoing research and multidisciplinary engagement in the evolving field of spinal infection management.

## 1 Introduction

Primary spinal infections, including conditions such as spondylodiscitis, vertebral osteomyelitis, and epidural abscess, represent a significant and growing challenge in clinical practice (Lener et al., 2018). Over the past decade, their incidence has increased, driven by factors such as an aging population and higher rates of immunosuppression (Kramer et al., 2023; Thavarajasingam et al., 2023a). This rise in cases highlights an urgent need for a standardized approach to the diagnosis and management of these complex conditions.

One of the most striking features of spinal infections is their heterogeneity. These infections vary widely in etiology, clinical presentation, and progression, from localized pain and mild instability to severe deformities, neurological deficits, and systemic complications such as sepsis. This variability complicates clinical decision-making, leading to inconsistencies in diagnosis and treatment strategies (Kramer et al., 2024a). The lack of clear, universally accepted criteria for surgical indications, treatment duration, and monitoring protocols further exacerbates these challenges, contributing to heterogeneity in outcomes.

Despite advancements in imaging and therapeutic options, the management of spinal infections is hindered by the absence of high-level evidence and a standard of care. Existing classification or scoring systems were based on monocentric datasets, often include overlapping or less impactful variables and while useful, lack the precision needed to fully capture the complexity of spinal infections and guide evidence-based decision-making and therefore has not reached broad implementation (Schömig et al., 2022; Pluemer et al., 2023; Quiceno et al., 2024; Hunter et al., 2022; Sircar et al., 2023; Akbar et al., 2011; Pola et al., 2017). This has resulted in ongoing uncertainty about which patients are most likely to benefit from surgical interventions compared to conservative approaches (Neuhoff et al.; Thavarajasingam et al., 2023b; Rutges et al., 2016).

To overcome the limitations of monocentric driven classifications or scores, there is a growing recognition of the need for precise and evidence-based framework, incorporating international experience and evidence. Such frameworks are not only essential for improving clinical decision-making but also foundational for advancing research (Laufer et al., 2013). Such a framework can enable the creation of well-defined cohorts for prospective trials, which are necessary to generate high-quality evidence and refine treatment protocols.

This manuscript is positioned as a groundwork paper to pave the way for future trials. It highlights the important variables in disease assessment to capture influencing factors on decision making and treatment outcomes. By providing a structured approach to stratifying patients, this pre-classification framework is intended to inform future trial design that test surgical indications and techniques, validate scoring systems, and ultimately improve outcomes in this increasingly prevalent and complex condition.

### 1.1 Assessment variables of spinal infection

The complexity of spinal infections requires the integration of multiple clinical, radiological, and laboratory findings to guide diagnosis and treatment decisions. Combining key elements of assessment and management of spinal infections it comes down to four core themes—Stability, Neurology, Infection, and Morbidity—that collectively encapsulate the key hypotheses central to treatment decision-making. Based on current evidence and expert consensus, the following factors are considered pivotal in spinal infection assessment (Kramer et al., 2025a).

1. Stability

Instability in spinal infections differs from other pathologies, such as trauma or neoplasms, in its rapid progression and distinct patterns of destruction. Infectious lesions typically arise at the vertebral endplate and spread to the surrounding motion segment, leading to ligamentous disruption and anterior vertebral defects. This pathophysiology underscores the importance of considering unique instability patterns in infectious conditions, as opposed to extrapolating criteria from other spinal pathologies.

Spinal stability is mainly evaluated by radiographic assessments supported by clinical signs like pain. The extend of vertebral body destruction seems to be a main factor of radiographic progression. Furthermore de novo kyphosis, scoliosis or translation indicate advanced instability that may necessitate surgical stabilization and restoration.

1.1. Pain

De novo pain syndromes, often the earliest and most prominent symptom of spinal infection, is a critical diagnostic hint that warrants further investigation. Mechanical pain, in particular, may indicate segmental instability, which can lead to progressive impairment or even immobilization (White and Panjabi, 1990). However, pain is not solely a marker of instability; severe pain syndromes like radicular pain, are also commonly caused by paravertebral or epidural abscesses. Careful differentiation between mechanical and radiating pain and correlating pain quality to the underlying causes helps tailoring management strategies in spinal infections.

1.2. Radiographic Signs

Magnetic Resonance Imaging (MRI) is widely recognized as the gold standard for diagnosing spinal infections, providing critical insights into soft tissue and bone involvement, epidural empyema, and the degree of instability or deformity. Complementary imaging modalities, such as CT scans, are essential for evaluating bony destruction and instability signs, while standing X-rays are frequently employed to detect deformities and dynamic instability. Together, these imaging techniques form the backbone of diagnostic evaluation and guide clinical decision-making (Kramer et al., 2025a).

**Fig. 1:**
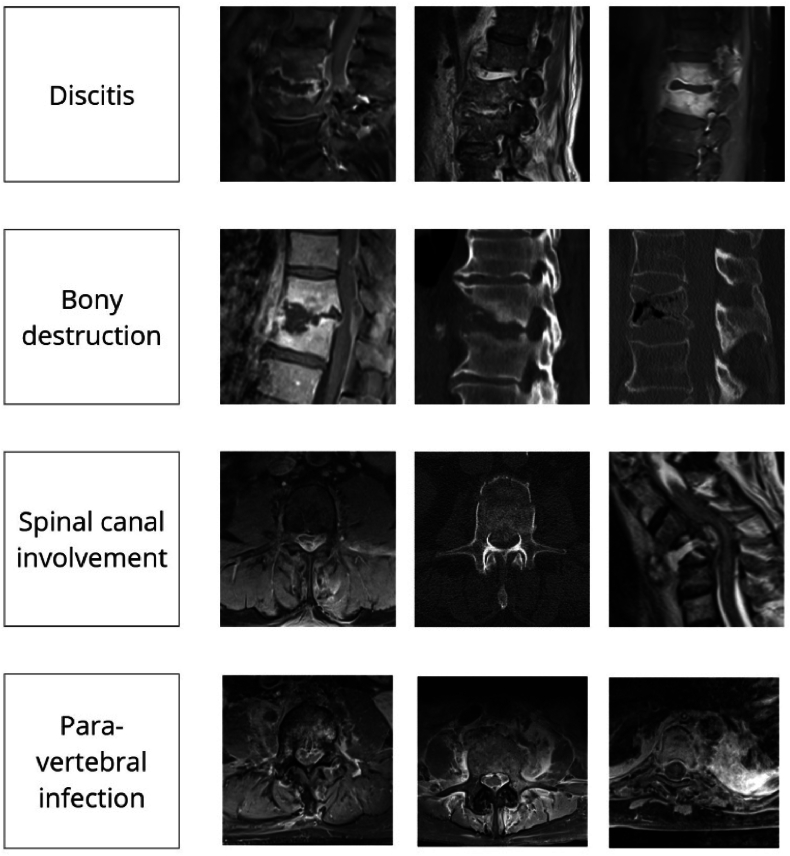
This figure presents representative MRI and CT images illustrating key radiological features associated with spinal infections. The first row shows discitis, characterized by signal alterations and destruction of the intervertebral disc space, typically with T2 hyperintensity and T1 hypointensity on MRI. The second row highlights bony destruction, demonstrating vertebral endplate erosion and collapse, as seen in both MRI and CT images. The third row illustrates spinal canal involvement, including epidural abscesses and neural compression visible in axial and sagittal views. The fourth row depicts paravertebral infection, showing inflammatory changes and abscess formation in the adjacent soft tissues, often enhancing with contrast on MRI.

Radiographic assessment includes several key factors that play a pivotal role in diagnosing and evaluating the severity of spinal infections (Kramer et al., 2025b). See Fig. 1 for imaging examples..

• Discitis, characterized by inflammation or infection of the intervertebral disc, manifests as a loss of disc height and structural integrity, with T2 hyperintensity and T1 hypointensity on MRI.
• Bony erosion, evident as irregularities or discontinuities in the endplate margins resembling moth-eaten necrosis, highlights the destructive impact of spondylodiscitis on vertebral bodies. Further osteonecrosis may lead to vertebral body destruction, with the extent of destruction being a critical variable for assessing kyphotic misalignment (Kramer et al., 2025b).
• Epidural abscesses, appearing as high T2 and low T1 signal intensity collections in the spinal canal with contrast enhancement, are often associated with neural compression, serving as major indicators for decompressive surgery.
• Paravertebral soft tissue reactions or abscesses are an important distinguishing feature, helping to differentiate spinal infections from aseptic degenerative conditions like erosive osteochondrosis.
• Although less common, involvement of posterolateral elements, such as facet joints, can contribute to further instability.
• De novo segmental angulation/kyphosis (>20°), scoliosis (>10°) or translation indicates advanced instability caused by significant mechanical disruption.

These radiographic findings collectively provide a comprehensive basis for evaluating the severity of spinal infections in respect of stability, neural compression and infectious collections for further tailoring clinical management.

The Spinal Instability Spondylodiscitis Score (SISS) and the Spinal Infection Treatment Evaluation (SITE) Score have recently been introduced to evaluate radiographic instability and assist in treatment decisions (Schömig et al., 2022; Pluemer et al., 2023). The SISS, derived from the Spinal Instability Neoplastic Score (SINS), focuses on assessing instability primarily through CT-based imaging, examining factors such as vertebral body collapse, pain, and deformity (Pithwa and Sinha Roy, 2023). While moderately effective in identifying gross instability, the SISS does not account for soft-tissue changes or systemic factors, limiting its applicability in complex cases. In contrast, the SITE Score integrates both CT and MRI findings, offering a more detailed and dynamic evaluation that includes early indicators like vertebral edema, endplate erosion, and posterolateral involvement. Additionally, the SITE Score emphasizes spinal canal stenosis with neural impingement, highlighting the critical overlap between instability and neurological risk. Despite their contributions to structuring the assessment of spinal infections, particularly in evaluating spinal instability, neither score has yet been validated as a tool to guide decision-making or precisely predict treatment failure in a clinical setting (Quiceno et al., 2024).

2. Neurology

Neurological deficits in spinal infections often arise from complex underlying causes, such as epidural abscesses, osseous canal involvement, spinal cord impingement due to kyphotic misalignment, translational deformity or the exacerbation of preexisting spinal canal stenosis. Solitary radiographic signs of neural compression may play an important role in surgical decision making to prevent imminent neurological deficits but clinical neurological impairment is the most decisive factor for surgical decompression.

A detailed neurological examination is crucial, as neurological deficits are a critical determinant in deciding the need for surgical intervention in spinal infections. Particularly new or progressive neurological deficits, such as radicular motor weakness, neurogenic bladder or bowel dysfunction, and spinal cord symptoms, profoundly impact functional outcomes and patient quality of life (Kramer et al., 2025a).

While specific classification systems exist for grading the extent of radiological neural compression in degenerative and oncologic cases, such a standardized grading system is lacking for spinal infections due to its heterogeneous nature (Bilsky et al., 2010; Schizas et al., 2010).

3. Infection

Infection control is a major goal of treatment to cure the disease. Acute/subacute, chronic or even aseptic forms of spinal infection exist, but to date there is no explicit definition of diagnostic criterias for degree of severity. Besides different pathogens exhibit distinct patterns in disease progression, systemic response, and antibiotic respondance (Hijazi et al., 2024). As such, effective treatment of spinal infections hinges on accurate pathogen identification to enable targeted antimicrobial therapy.

Psoas and epidural abscesses can further complicate infection control, as their encapsulated nature shields pathogens from immune surveillance, often necessitating drainage.

Debridement combined with antibiotic therapy plays a crucial role, particularly in critically ill or immunocompromised patients. Despite their elevated surgical risk, these patients may still require intervention to reduce the infectious load and support recovery. Drainage or surgical debridement may be required to mitigate complications and prevent systemic dissemination.

3.1. Pathogens

Common pathogens include *Staphylococcus aureus* (including MRSA), *Streptococcus* species, and gram-negative bacteria such as *Escherichia coli* (Park et al., 2019; Berbari et al., 2015). Immunocompromised patients and intravenous drug users may present with atypical or polymicrobial infections, including *Pseudomonas aeruginosa*, fungi, or mycobacteria (Bilolikar et al., 2024). Understanding the microbiological profile is vital not only for treatment selection but also for anticipating disease progression and potential complications. Despite its critical role, the behavior of specific pathogens in spinal infections—including their impact on disease progression and treatment outcomes—remains insufficiently studied, underscoring the need for further research in this area.

3.2. Laboratory Parameters

Among laboratory markers, C-reactive protein (CRP) has emerged as the most reliable parameter for diagnosing and monitoring spinal infections. CRP's sensitivity, dynamic response to inflammation, and its association with disease severity make it indispensable in clinical evaluation (Kramer et al., 2025a). Elevated CRP levels are incorporated into prognostic tools, where CRP plays a central role in identifying patients at higher risk of complications (Hunter et al., 2024; Patel et al., 2014). Those patients exceeding thresholds of 140–200 mg/L, have been strongly linked to severe disease and adverse outcomes, including higher mortality rates (Lener et al., 2022; Heuer et al., 2022; Kramer et al., 2024b).

By contrast, traditional markers like white blood cell count (WBC) and erythrocyte sedimentation rate (ESR) are less sensitive and specific, with limited prognostic utility (Kramer et al., 2025a). Procalcitonin, while showing promise in sepsis settings, is not yet widely validated for routine use in spinal infections (Jeong et al., 2015; Santagada et al., 2022).

3.3. Systemic Immune Response Syndrome (SIRS)

SIRS is a rare but devastating complication in the context of spinal infections, indicating a systemic reaction to local infection and often heralding a severe disease course (Kramer et al., 2024b). It is characterized by clinical signs such as fever or hypothermia, tachycardia, tachypnea, and leukocytosis or leukopenia, reflecting widespread inflammation. In spinal infections, the presence of SIRS is associated with a significantly higher risk of morbidity and mortality, especially in elderly or immunocompromised patients (Ziarko et al., 2023).

4. Morbidity

Morbidity is a modifying factor that influences all aspects of spinal infection management. Systemic health factors, including age, frailty, and comorbidities, profoundly affect decision-making, treatment outcomes, and overall prognosis. These characteristics, while increasing surgical risks, should not preclude intervention. Instead, they call for tailored approaches to balance risks and benefits effectively. For instance, older or immunosuppressed patients may require extended antibiotic regimens and minimally invasive surgical techniques.

4.1. Comorbidities and immunosuppression

Immunosuppressive comorbidities, including diabetes, renal or hepatic failure and prolonged immunosuppressive medication, are critical contributors to the genesis and progression of primary spinal infection (Tsantes et al., 2020; Jochimsen et al., 2024). These conditions impair the body's ability to combat bacterial infections effectively, increasing susceptibility to spinal infections and complicating outcomes. and influences recovery (Berbari et al., 2015; Ukon et al., 2025).

However, it is not evident that specific comorbidities consistently stand out in spinal infections; instead, general comorbidity indices like the Age-Adjusted Charlson Comorbidity Index (ACCI) and the American Society of Anesthesiologists (ASA) Score have demonstrated utility in predicting unfavorable outcomes, such as mortality and surgical risks (Lener et al., 2022). Mortality risk score systems for spinal infections like MSI-20 and HSAS Score rely on CRP alongside parameters like age, comorbidities and creatinine levels to predict mortality (Lener et al., 2022; Heuer et al., 2022). As variability and risk of bias is high, theses Scores would require validation using large-scale, multicenter datasets with homogeneous treatment groups to achieve broader applicability and greater precision in clinical decision-making.

4.2. Age and frailty

The rising incidence of spinal infections among the elderly, makes this population a focal point for research and clinical interest (Kramer et al., 2023; Thavarajasingam et al., 2023a; Aoyama et al., 2023). Older patients with spinal infections often present with multiple immunosuppressive comorbidities, such as diabetes, chronic kidney disease, and cardiovascular disease, which significantly complicate treatment and recovery. Managing spinal infections in this population necessitates a delicate balance between aggressive treatment and careful consideration of the patient's overall health status (Lee et al., 2021).

Evidence indicates that frailty, a dynamic, age-associated decline in physiological reserve and resilience to stressors, is linked to increased risks of postoperative adverse events, including mortality, prolonged hospitalizations, and suboptimal functional recovery (Alas et al., 2020). Current frailty assessment models, such as the modified frailty index (mFI) offer promising avenues for risk stratification, particularly in predicting postoperative complications in spinal surgery (Camino-Willhuber et al., 2023; Kweh et al., 2023). However, their applicability in spinal infection management remains unclear. Further research is needed to identify and validate an optimal frailty tool for use in this context.

4.3. Intravenous drug abuse

Beyond the elderly population, intravenous drug abusers (IVDA) represent another unique and challenging subgroup in spinal infection management (Bilolikar et al., 2024; Reid et al., 2019; Nishizawa et al., 2025). Although typically younger, IVDA patients face significant obstacles due to higher rates of mental health issues, recurrent bacteraemia, and noncompliance with treatment protocols (Bilolikar et al., 2024; Tavolaro et al., 2022). These factors not only complicate postoperative care but also elevate the risk of treatment failure, requiring tailored management strategies that address the psychosocial and medical complexities inherent in this group.

### 1.2 Classification of spinal infections

**Fig. 2:**
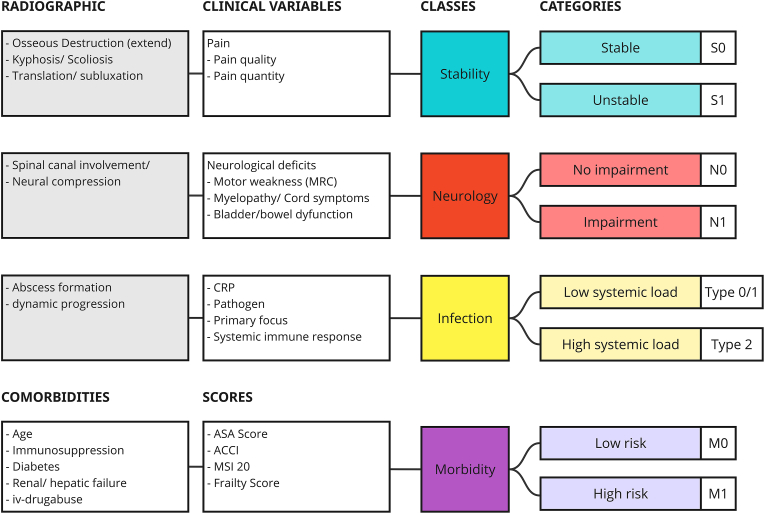
This figure presents the SNIM classes, providing the variables for the assessment of spinal infections based on four core domains: Stability, Neurology, Infection, and Morbidity. Each domain integrates relevant radiographic findings, clinical variables, and is stratified into clinically meaningful categories to support consistent evaluation and treatment planning. Stability is assessed through radiographic indicators such as vertebral body destruction, kyphotic or scoliotic deformity, and translational instability, along with clinical pain characteristics. Neurology captures the presence of neural compression on imaging as well as clinical deficits, including motor weakness, myelopathy, or bladder and bowel dysfunction. The Infection domain evaluates systemic infectious burden by considering radiographic features like abscess formation, laboratory parameters such as C-reactive protein (CRP), pathogen identification, and evidence of systemic immune response. Morbidity accounts for patient-specific risk factors, including age, comorbidities such as diabetes or renal failure, immunosuppression, and intravenous drug use. Established scoring systems such as the ASA Score, the Age-adjusted Charlson Comorbidity Index (ACCI), the MSI-20, and frailty indices are included to stratify patients into low- or high-risk categories.

Fig. 2 illustrates the variables, classes and categories.

By addressing stability, neurology, infection, and morbidity, this system captures the multifaceted nature of spinal infections and lays the foundation for a unified classification framework. As displayed in Fig. 2, each core element integrates clinical, radiographic, and laboratory findings, offering a comprehensive approach to spinal infection assessment. However, the relative significance of each variable in decision-making, disease progression, and treatment outcomes remains to be explored. Consequently, this classification system refrains from assigning specific grades to individual variables at this stage. Future efforts will focus on validating this framework through prospective studies, which will aim to refine and subdivide each element into distinct grading scales. Such an approach has the potential to enhance patient stratification, enabling tailored treatment strategies that align with individual clinical profiles. Ultimately, this evolution of the classification system may pave the way for more precise patient selection and robust, evidence-based decision-making in the management of spinal infections.

## 2 Management of spinal infections

1. Pathogen identification and antibiotic treatment

Antibiotic treatment remains the cornerstone of spinal infection management. The selection of antibiotics should be tailored to the identified pathogen whenever possible. In cases with negative microbiological results, empiric antibiotic regimens should be initiated, focusing on coverage of the most common pathogens, including *Staphylococcus*, Enterobacteria, and Streptococci (Berbari et al., 2015). The duration of antibiotic therapy varies based on the severity and complexity of the infection. Less severe cases, where the infection is well-controlled and there is no extensive tissue destruction or systemic involvement, can often be managed with a shorter antibiotic course of six weeks (Park et al., 2016). However, more complex cases, such as those involving resistant pathogens, immunosuppressed patients, or older individuals with multiple comorbidities, frequently require prolonged therapy lasting up to 12 weeks (Kramer et al., 2025a).

Accurate microbiological diagnosis is essential for guiding targeted antibiotic therapy (Stangenberg et al., 2021). Blood cultures, ideally drawn before initiating antibiotic therapy, are a first diagnostic step in all cases of spinal infection, including patients without fever (Kramer et al., 2025a). Despite their importance, the positivity rate for blood cultures varies widely, depending on factors such as prior antibiotic use and the underlying microbial etiology (Nolla et al., 2002; Hijazi et al., 2023).

In instances where blood cultures fail to identify a pathogen, a CT-guided biopsy of the affected disc, vertebral body or paravertebral abscess is recommended (Kramer et al., 2025a; Malik et al., 2024; Nguyen et al., 2021) but surgical debridement and intraoperative sample collection maximizes the chances of pathogen identification, providing essential information for tailoring antimicrobial therapy (Stangenberg et al., 2021; Hijazi et al., 2023). Development of more sensitive pathogen detection methods like sequencing, targeted and resistance-aware antimicrobial therapies may further refine medical treatment.

Beyond identifying the direct cause of the infection, locating a potential primary focus of infection is equally important to address the source and prevent recurrence. Comprehensive evaluation should include imaging such as CT of the thorax, abdomen, and pelvis to identify possible septic foci. A cardiac ultrasound, particularly transesophageal echocardiography (TEE), is recommended to rule out infective endocarditis, a common associated condition. Additional investigations should include dental and skin examinations, urinary analysis, and evaluation of any indwelling devices or implants, as these are frequent sources of hematogenous spread (Kramer et al., 2025a).

2. Indication and timing of surgical management

Our recent meta-analysis study suggested that early surgical treatment of spinal infections can reduce mortality by up to 39 % and significantly shorten hospital stay (Thavarajasingam et al., 2023b). Although the precise mechanisms behind this benefit remain unclear, proposed explanations include earlier mobilisation, reduction of mechanical stress on the infected segment, and decreased infectious burden. These assumptions, while supported by clinical reasoning and analogies to other spinal pathologies, were further reinforced by our comparative analysis, which showed favorable outcomes with predominantly combined anterior–posterior approaches—where stabilization and debridement played a central role (Neuhoff et al., 2025).

Accordingly, early treatment of spinal instability is considered a key indication for surgical intervention. Beyond radiological signs, persistent or severe mechanical pain—particularly under axial loading—is often a decisive clinical indicator, as prolonged immobilization is a known risk factor for increased morbidity and mortality (Pithwa and Sinha Roy, 2023). In the long term, stabilization may help prevent the progression of spinal deformity and its associated disability and persisting pain (Kramer et al., 2025b; Srinivasan et al., 2014; Neuhoff et al., 2024).

Robust evidence exists for early surgical decompression in cases of neurological compromise due to trauma, degenerative disease, or neoplasms, but data on spinal infections remain limited Nevertheless, in cases of spinal epidural abscess (SEA), two studies from 2014 demonstrated a higher failure rate of medical management (up to 41 %)—often accompanied by deterioration on the ASIA motor scale and persistent deficits—compared to early surgical intervention (Patel et al., 2014; Ghobrial et al., 2014). Consequently, the EANS Spine Section Delphi consensus considered early surgical decompression essential in the presence of new or progressive neurological deficits, such as radicular motor weakness (less than grade 4/5), spinal cord symptoms, or neurogenic bladder or bowel dysfunction, even in the absence of definitive randomized evidence for infectious etiologies (Kramer et al., 2025a; de Leeuw et al., 2018).

Patients presenting with Systemic Inflammatory Response Syndrome (SIRS) constitute a particularly high-risk subgroup requiring individualized management strategies. Data from the 2SICK study highlight the benefits of delayed surgical intervention in these cases, identifying an optimal window of 10–14 days post-admission. This approach allows for stabilization of systemic parameters and mitigates perioperative risks such as circulatory collapse or hematogenous bacterial dissemination. In this cohort, delayed surgery significantly reduced mortality compared to both early surgical and conservative treatment approaches (Kramer et al., 2024b; Al-Afif et al., 2023).

3. Surgical techniques

**Fig. 3:**
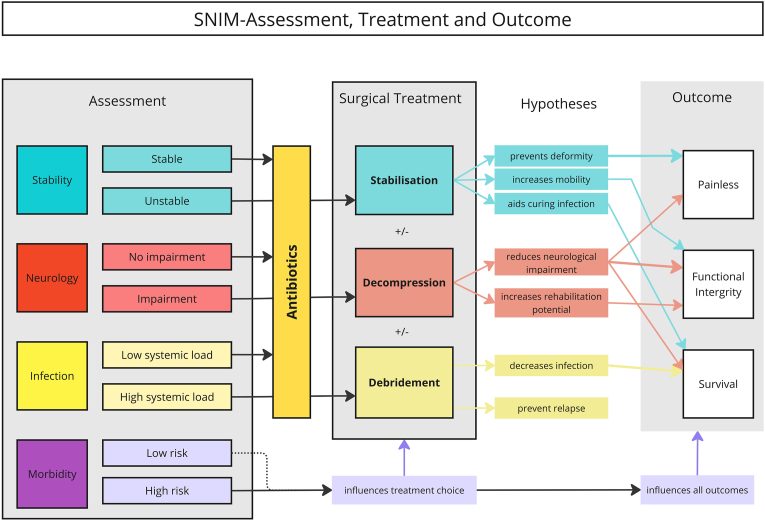
This diagramm illustrates the interplay between SNIM domains and surgical modalities, offering hypotheses to be tested in future trials: The process begins with a structured assessment across four domains - Stability, Neurology, Infection, and Morbidity - which collectively inform both the need for and the nature of surgical treatment interventions. All patients receive antibiotic therapy as a cornerstone of infection management. Depending on the individual SNIM profile, surgical treatments may include stabilization, decompression, and/or debridement. Each surgical modality is associated with specific hypotheses regarding its impact on different outcomes, like pain, disability or death.

As illustrated in Fig. 3 the surgical treatment of spinal infections is centered around three key techniques: stabilization, decompression, and debridement. Each technique addresses specific aspects of the disease, often working in combination to provide comprehensive care. However, while the rationale for these approaches is well-established, high-level evidence supporting their efficacy in improving both short- and long-term outcomes remains limited. This gap has led to the formulation of specific hypotheses for each technique, emphasizing their potential roles in improving patient outcomes. These hypotheses underscore the need for large prospective trials to validate the role of surgical interventions in spinal infection management and to optimize treatment strategies.

3.1. Stabilization

Stabilization plays a crucial role in restoring spinal integrity, alleviating mechanical pain, and preventing the progression of deformities. Posterior stabilization using pedicle screws and rods is the most common approach at the thoracolumbar spine, although anterior column reconstruction can be used for extensive vertebral body destruction (Neuhoff et al., 2024). In the cervical spine, both anterior and posterior approaches can be appropriate, depending on the level involved and the extent of osseous compromise.

The choice of surgical technique—such as percutaneous versus open approaches, or posterior-only versus combined anterior–posterior instrumentation—is typically guided by the degree of bony destruction, bone quality, and the patient's overall clinical condition. To date, no high-level evidence demonstrates clear superiority of one technique over another in terms of mortality, infection control, or implant failure. However, minimally invasive approaches have been associated with lower intraoperative blood loss and reduced rates of wound-related complications compared to open procedures. (Schatlo et al., 2023; Cabrera et al., 2023).

Testable Hypothesis.

• Surgical stabilization of unstable segments in spinal infection reduces short-term disability and morbidity by enabling early mobilisation.
• Surgical stabilization of unstable segments in spinal infections reduces long-term disability and chronic pain by preventing deformity progression.
• Surgical stabilization aids in curing the infection by reducing mechanical stress on the infected motion segment.

3.2. Decompression

Decompression is essential for addressing neurological deficits caused by the infection, such as motor weakness, sensory impairment, or bowel and bladder dysfunction. It is particularly critical in cases of epidural empyema compressing neural structures. Surgical techniques for decompression depend on the location of the lesion. Thoracolumbar lesions are typically managed with posterior decompression, while cervical infections may be decompressed anteriorly or posteriorly.

Testable Hypothesis.

• Early decompression in cases of neurological deficits leads to better functional outcomes and reduces the risk of permanent disability.
• Early decompression in cases with no clinical neurological deficit but radiographic neural compression prevents functional impairment and disability.

3.3. Debridement

Debridement focuses on reducing the infectious burden, promoting healing, and creating a favorable environment for antibiotic therapy. Surgical debridement involves removing infected and necrotic tissues or bone, often through minimally invasive or open approaches, followed by irrigation of the surgical field with saline or antiseptic solutions. By effectively reducing the infectious load, debridement may facilitate faster resolution and prevent relapse.

Testable Hypothesis.

**Fig. 4:**
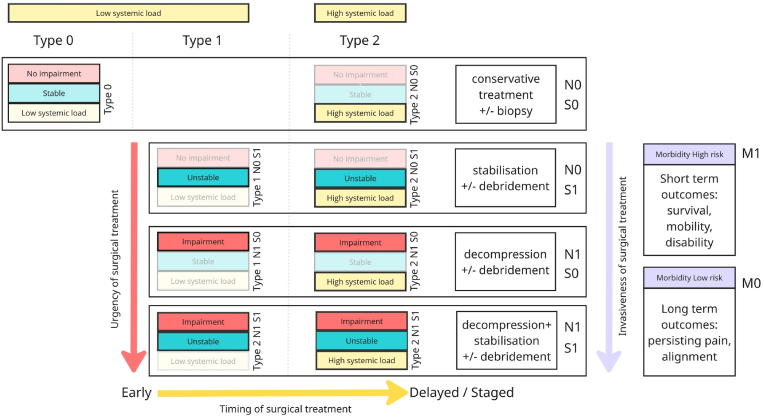
Conceptual treatment algorithm based on SNIM stratification – a working model to guide discussion and study design: This figure presents a treatment algorithm for spinal infections based on the SNIM framework, stratifying patients into subgroups according to the presence of neurological impairment (N1), spinal instability (S1), and the severity of systemic infection (Type 0–2). The urgency for surgical intervention increases from top to bottom, as indicated by the red arrow. Type 0 patients with stable clinical and radiological findings may be managed conservatively. Type 1 involves localized infections requiring targeted surgical procedures, either decompression or stabilization or both. Type 2 reflects advanced disease with systemic infection and frequently requires multimodal medical and surgical strategies. Morbidity status (M0 = low risk, M1 = high risk) serves as a treatment modifier: in low-risk patients, long-term outcomes guide management, potentially justifying more extensive procedures. In contrast, high-risk patients require a focus on short-term outcomes such as survival and early mobilization, favoring minimally invasive approaches to limit physiological stress. This algorithm reflects expert interpretation of available evidence. It is not intended as a validated clinical decision tool but as a proposal for future evaluation.

• Combined early surgical debridement and targeted antibiotic therapy reduce infection relapse rates, systemic complications, and mortality.

4. Treatment algorithm
  Fig. 4 presents a treatment algorithm for spinal infections based on the SNIM framework. Patients are stratified into Type 0, Type 1, and Type 2, with the distinction between Type 1 and Type 2 based on the severity of systemic infection. The urgency of surgical treatment increases vertically, while the need for systemic restoration prior to surgery increases from left to right.

• Type 0 patients have no neurological deficits (N0), a stable spine (S0), and a low systemic infection burden, representing local infections. These cases may typically be managed conservatively with blood cultures and/or image-guided tissue sampling for microbiological diagnosis, followed by targeted antibiotic therapy. Close monitoring of clinical status, inflammatory markers, and serial radiological imaging is essential to detect progression, secondary instability, or relapse (Kramer et al., 2025b; Berbari et al., 2015).
• Type 1 patients present with localized spinal infections and involvement of one additional SNIM domain. In these cases, early surgical intervention has been associated with improved survival and preservation of neurological function (Neuhoff et al.; Thavarajasingam et al., 2023b).
• Type 2 patients present with a high systemic infectious burden, reflecting a more advanced stage of disease that typically requires multimodal treatment. In the presence of systemic dysfunction, a carefully balanced, delayed or staged approach may be appropriate to allow for systemic restoration and to reduce perioperative risk (Kramer et al., 2024b; Al-Afif et al., 2023).
• N1 S0: Patients with neurological deficits but preserved spinal stability—such as those with simple or multilevel epidural abscesses—are candidates for microsurgical or endoscopic decompression, often combined with spinal canal lavage (Patel et al., 2014; de Leeuw et al., 2018; Moschos et al.).
• N0 S1: Patients with mechanical instability but no neurological deficits may be treated with simple stabilization. They may require combined approaches, including segmental stabilization and debridement via abscess drainage, disc removal, or even osseous resection (Akbar et al., 2011; Neuhoff et al., 2024; Schatlo et al., 2023; Cabrera et al., 2023).
• N1 and S1: Patients with both neurological deficits and instability represent the most vulnerable subgroup, with elevated risks for mortality and persistent disability and pain. Combined decompressive/stabilization procedures are required to reduce mechanical stress on the neural elements and spinal motion segment. Timely decompression is crucial, combined with thorough debridement of infected tissue to relieve neural structures.
  Morbidity status (M0 = low risk, M1 = high risk) serves as a key modifying factor in treatment planning.
• In low-risk patients (M0), the therapeutic goal is to optimize long-term outcomes, which could include restoring sagittal balance, reducing stress on adjacent segments, and minimizing the risk of relapse. Extended procedures, including multilevel instrumentation, combined approaches, or osteotomies, may be warranted.
• In high-risk patients (M1), treatment may prioritize short-term goals such as survival, early mobilization and prevention of severe disability. In this group, minimally invasive and/or quick techniques could be preferred avoiding extensive tissue trauma and excessive blood loss, that might compromise the patient's already limited systemic inflammatory reserves.

## 3 Conclusion

This manuscript and its preparatory work aim to provide a conceptual foundation for advancing the assessment and management of spinal infections. While existing classification systems and scores have focused on individual disease aspects—such as surgical approach, spinal stability, or mortality risk—the SNIM framework is the first to integrate the four core domains of Stability, Neurology, Infection, and Morbidity into a unified structure. It remains a preliminary classification framework in which thresholds and limits must be defined and tested through clinical trials.

This position paper also seeks to support the design of clinical trials by outlining testable hypotheses regarding key surgical modalities (stabilization, decompression, and debridement) while addressing a major source of variability: patient selection. In addition to surgical technique, the timing of intervention emerges as a potentially critical determinant of outcome, particularly in systemically compromised patients. The definition of meaningful treatment goals—whether focused on deformity correction, prevention of disability, or simply survival—should be adapted to the patient's overall clinical status and comorbid burden.

To this end, we introduce a treatment algorithm based on SNIM stratification. This working model is intended to support structured discussion, reduce clinical heterogeneity in trial design, and encourage the development of personalized treatment strategies. It incorporates the modifying role of morbidity and proposes a temporal dimension for surgical timing. While the algorithm reflects current expert interpretation of the literature and emerging clinical patterns, it is not a prescriptive tool and requires evaluation in future studies.

By framing these conceptual tools and hypotheses, this position paper aims to stimulate collaborative, multidisciplinary efforts toward more consistent, evidence-informed care in spinal infection—a complex and increasingly prevalent clinical challenge.

## Declaration of competing interest

The authors declare that they have no known competing financial interests or personal relationships that could have appeared to influence the work reported in this paper.
